# Atherogenic index of plasma is related to coronary atherosclerotic disease in elderly individuals: a cross-sectional study

**DOI:** 10.1186/s12944-021-01496-8

**Published:** 2021-07-11

**Authors:** Haomin Huang, Xiaolong Yu, Lamei Li, Ganwei Shi, Feng Li, Jianqiang Xiao, Zhihua Yun, Gaojun Cai

**Affiliations:** 1 Department of Cardiology, Wujin Hospital Affiliated with Jiangsu University, The Wujin Clinical College of Xuzhou Medical University, Changzhou City, Jiangsu Province China; 2 Department of Ultrasound, Wujin Hospital Affiliated with Jiangsu University, The Wujin Clinical College of Xuzhou Medical University, Changzhou City, Jiangsu Province China; 3 Department of Clinical Laboratory, Wujin Hospital Affiliated with Jiangsu University, The Wujin Clinical College of Xuzhou Medical University, Changzhou City, Jiangsu Province China

**Keywords:** Atherogenic index of plasma, Coronary atherosclerotic disease, Elderly, Lipid metabolism, Atherosclerosis, Cross-sectional study

## Abstract

**Background:**

Dyslipidaemia plays an important role in coronary atherosclerotic disease (CAD). The relationship between the atherogenic index of plasma (AIP) and CAD in elderly individuals was explored in this study.

**Methods:**

Elderly individuals (age ≥ 65 years) who underwent coronary angiography from January 2016 to October 2020 were consecutively enrolled in the study.

**Results:**

A total of 1313 individuals, including 354 controls (non-CAD) and 959 CAD patients, were enrolled. In univariate analysis of all populations, the adjusted AIP (aAIP) in the CAD group was 1.13 (0.96, 1.3), which was significantly higher than that in the controls [1.07 (0.89, 1.26)]. However, in subgroup analyses, this phenomenon was only present in males. In addition, further study showed that aAIP was positively related to CAD severity. In binary logistic regression analyses, after adjusting for sex, age, smoking status, primary hypertension (PH), type 2 diabetes mellitus (T2DM), heart rate (HR), white blood cell (WBC) and platelet (PLT), AIP remained independently related to CAD in elderly individuals and was superior to traditional and other nontraditional lipid indices. Subgroup analyses showed that AIP independently influenced CAD risk in males. Ultimately, sensitivity analyses were performed excluding all coronary emergencies, and the final results were similar.

**Conclusions:**

AIP was positively related to the risk and severity of CAD in elderly individuals and was superior to traditional and other nontraditional lipid profiles. However, this association only exists in elderly males.

**Supplementary Information:**

The online version contains supplementary material available at 10.1186/s12944-021-01496-8.

## Background

With the accelerating pace of modern life and the development of social economics, people limit physical activity and eat more fat than before, resulting in coronary atherosclerotic disease (CAD), which is the primary cause of death worldwide [1, 2]. There are approximately 11 million CAD patients in China [3]. Among all casualties, elderly individuals bear the majority of the CAD burden [4, 5]. In addition, as life expectancy has increased, elderly individuals will gradually become a large part of the whole population, and approximately one-fifth of the world’s population will be older than 65 years of age by 2030 [6].

Elevated low-density lipoprotein cholesterol (LDL-C) increases the risk of CAD, but the risk of cardiovascular events remains after standardized lipid-lowering therapy [7]. Therefore, exploring a comprehensive blood lipid index that can better predict and evaluate CAD has become a research hotspot.

Various comprehensive lipid indices have been previously identified as associated with CAD. Among them, atherogenic index of plasma (AIP), which is expressed as logarithmic transformation of the quotient of triglycerides (TG) divided by high-density lipoprotein cholesterol (HDL-C) [lg(TG/HDL-C)], is a new reliable and predictable CAD index that has been verified in various populations, including Chinese Han people [8], very early onset CAD patients (≤35 years of age) [9] and type 2 diabetes mellitus (T2DM) patients after stent implantation [10]. The ability of AIP to predict CAD was stronger than other atherosclerosis indices, such as non-high-density lipoprotein cholesterol (non-HDL-C), which is calculated as TC minus HDL-C (TC - HDL-C); LDL-C/HDL-C; non-HDL-C/HDL-C, which is known as the atherogenic index (AI); and total cholesterol (TC) * TG * LDL-C/HDL-C, which is known as the lipoprotein combine index (LCI).

However, whether AIP could successfully predict the presence of CAD remains controversial [11–13]. Moreover, a previous study showed that elderly patients had obviously different clinical characteristics than young patients with acute myocardial infarction [14]. Whether AIP is related to CAD risk in elderly individuals has not been verified. In view of this information, this hospital-based cross-sectional study was conducted to explore the correlation between AIP and CAD in elderly individuals.

## Methods

### Participants

**Fig. 1:**
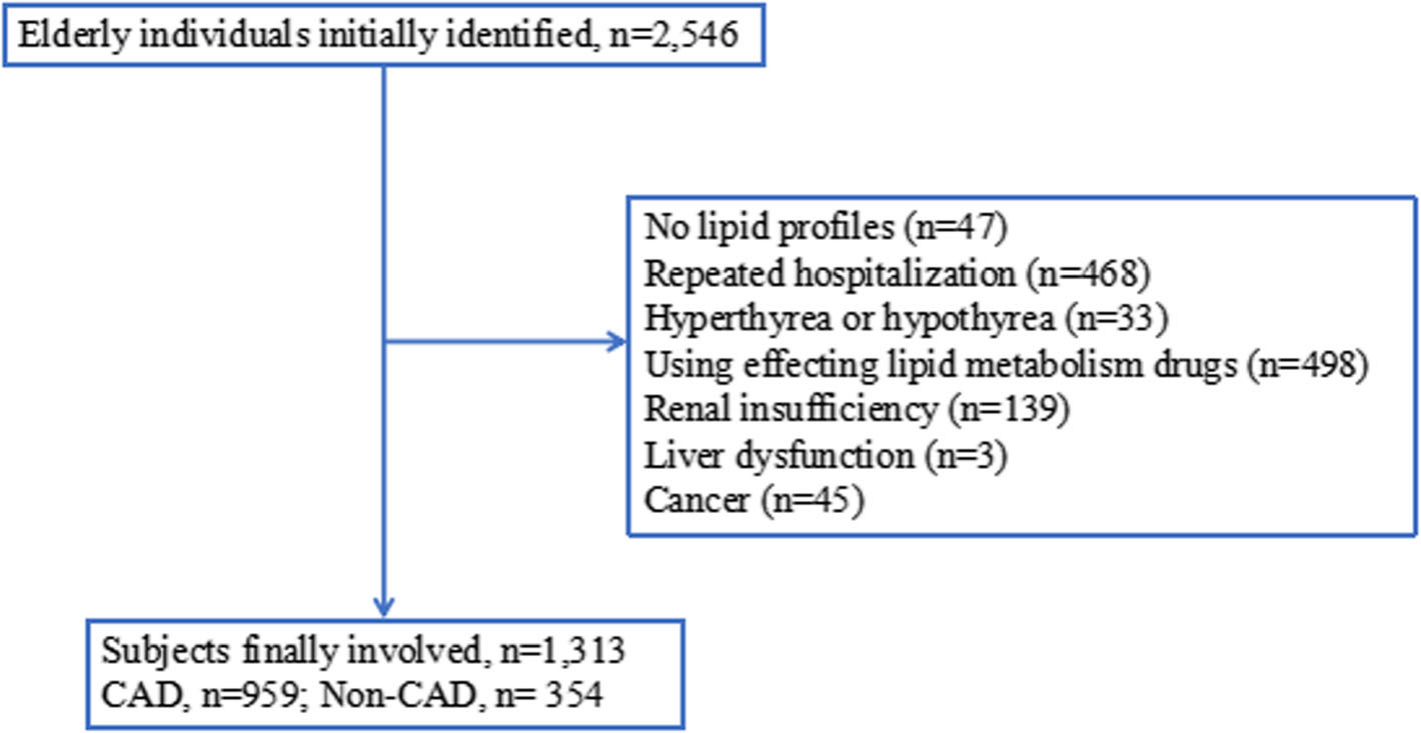
Flow chart describing the procedure of subjects involved in this study

Elderly individuals (≥65 years of age) who underwent coronary angiography (CAG) in a third-tier general hospital from January 2016 to October 2020 were consecutively selected. The exclusion criteria were as follows: 1) individuals with serious liver dysfunction [alanine aminotransferase (ALT) ≥200 U/L] or renal insufficiency [estimated glomerular filtration rate (eGFR) < 45 ml/min/1.73 m^2^], hyperthyroidism or hypothyroidism; 2) individuals using lipid metabolism drugs; 3) individuals with a history of cancer; and 4) individuals with repeated CAG and incomplete lipid profile data. Finally, a total of 1313 elderly participants, including 354 non-CAD controls and 959 CAD patients, were enrolled in this study. Figure 1 presents the flowchart outlining the study.

This study adhered to the tenants of the Declaration of Helsinki and was approved by the Institutional Ethics Committee of Wujin Hospital (Ethics approval number: 201606). Because this cross-sectional study was retrospectively designed, written informed consent was not needed.

### Diagnostic criteria

All subjects were examined by CAG via radial or femoral using the Judkin technique, and the CAG images were assessed by two experienced cardiologists who were blinded to the study. World Health Organization diagnostic criteria were used to diagnose CAD with at least one main coronary artery exhibiting ≥50% stenosis [15], and the severity of CAD was evaluated according to the number of main coronary arteries ≥50% stenosis. The control group was defined as individuals with main coronary artery stenosis < 50%. Primary hypertension (PH) and T2DM were defined according to the characteristics described in a previous study [16]. In short, PH was defined as repeated systolic blood pressure (SBP) ≥140 mmHg and/or diastolic blood pressure (DBP) ≥90 mmHg at least three times on different days or previously diagnosed PH. T2DM was defined as two measures of fasting blood glucose ≥7.0 mmol/L and/or random glucose level ≥ 11.1 mmol/L plus symptoms or previously diagnosed T2DM. A smoker was defined as an individual who smokes more than ten cigarettes per day, and a drinker was defined as an individual who intakes more than 40 ml of alcohol per day.

### Data collection

After fasting for 12 h, venous blood was obtained from each participant. General data, including sex, age, height, weight, SBP, DBP, heart rate (HR), PH, T2DM, smoking status, and drinking status, were extracted from medical records. Biochemical parameters, including white blood cell (WBC), platelet (PLT), serum uric acid (SUA), eGFR, and lipid profiles, were analysed using an automated biochemical analyser.

Body mass index (BMI) was expressed as the quotient of weight divided by height squared (weight/height^2^). To avoid negative AIP values, TG/HDL-C was multiplied by 10, and then the logarithmic value was obtained as the adjusted AIP (aAIP) [lg(TG/HDL-C*10)].

### Statistical analyses

SPSS 22.0 software (SPSS Inc., IL, USA) and Stata 12.0 software were used to analyse all data. Missing values were replaced by the median value. Continuous variables distributed normally were expressed as the means ± standard deviation and compared using Student’s t-tests, and those distributed abnormally were expressed as median and interquartile range (IQR) and compared using Mann-Whitney U test. The Kolmogorov-Smirnov test was used to evaluate the normality of the data. Categorical variables were assessed by Chi-square test. The correlations between aAIP and age, BMI, SUA and LDL-C were performed using Spearman correlation analysis. All variables were explored at four aAIP levels base on the median and IQR. The relationship between aAIP and the risk of CAD was explored using binary logistic regression analyses expressed as odds ratio (OR) with 95% confidence intervals (95% CI). In the model of all individuals, OR was adjusted for sex, age, smoking status, PH, T2DM, HR, WBC and PLT. In the elderly male model, OR was adjusted for alcohol consumption, T2DM, WBC, PLT, and SUA. In the elderly female model, OR was adjusted for age, PH, T2DM, HR and WBC. A two-sided *P-*value < 0.05 was considered statistically significant.

## Results

### Clinical characteristics of all participants

**Fig. 2:**
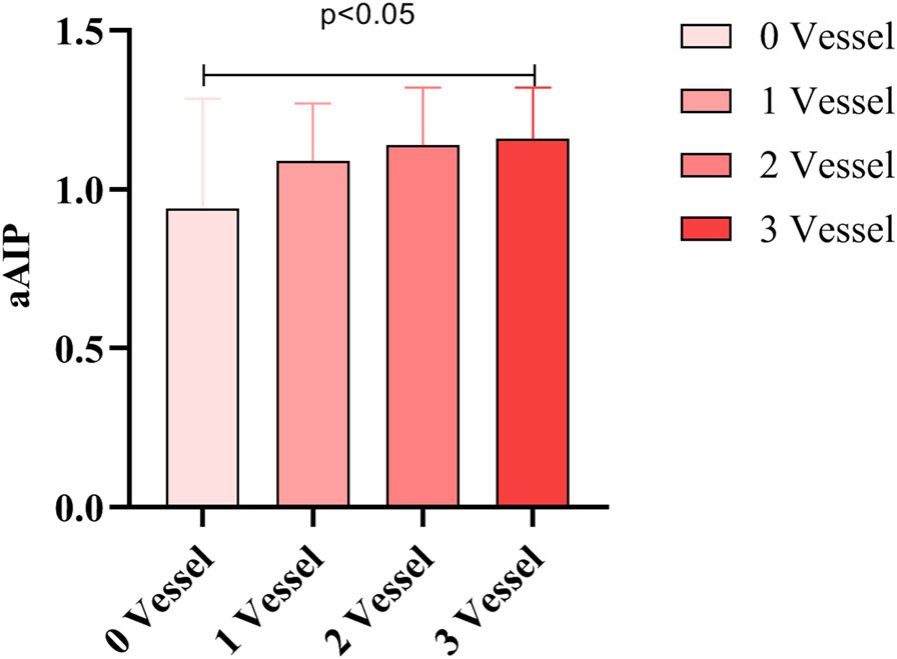
The relationship between aAIP and the severity of CAD

As shown in Table 1, it was obvious that age and the prevalence of males were higher in the CAD group compared with the controls [age, 71 (68, 75) vs 70 (67, 74), *P* < 0.05; males, 63.6% vs 51.7%, *P* < 0.001]. Elderly individuals with CAD had a significantly higher prevalence of smoking, PH and T2DM compared with controls (all *P* < 0.05). In addition, CAD individuals had higher levels of HR, WBC, PLT, TC, LDL-C, aAIP, non-HDL-C, LDL-C/HDL-C, AI and LCI and had lower levels of HDL-C than those of the controls (all *P* < 0.05). As for aAIP, it’s levels continually increased with the aggravation of CAD showed in Fig. 2. Nevertheless, the proportion of drinkers and BMI, SBP, DBP, eGFR, SUA and TG levels were not significantly different between the two groups (all *P* > 0.05).

**Table 1:** Clinical characteristics of whole participants

Characteristics	Control group(***n*** = 354)	CAD group(***n*** = 959)	***P***
Male, n(%)	183 (51.7)	610 (63.6)	< 0.001
Age, year	70 (67, 74)	71 (68, 75)	0.001
BMI, kg/m^2^	24.57 (22.67, 27.10)	24.39 (22.41, 26.42)	0.067
Smoker, n(%)	86 (24.3)	316 (33)	0.003
Drinker, n(%)	45 (12.7)	108 (11.3)	0.467
PH, n(%)	248 (70.1)	788 (78.1)	0.003
T2DM, n(%)	79 (22.3)	313 (32.6)	< 0.001
SBP, mmHg	139 (128, 150)	140 (127, 152)	0.215
DBP, mmHg	80 (71, 86)	80 (72, 89)	0.192
HR, BPM	70 (66, 80)	72 (67, 80)	0.031
Laboratory parameters			
WBC, 10^9^ /L	5.97 (5, 7.06)	6.79 (5.52, 8.43)	< 0.001
PLT, 10^9^ /L	187.5 (155, 228.25)	196 (162, 236)	0.016
eGFR, ml/min	75.09 (63.54, 89.23)	74.24 (60.95, 87.13)	0.179
SUA, umol/L	347.3 (291, 426.2)	347 (286.6, 415)	0.369
TC, mmol/	4.28 (3.68, 4.91)	4.42 (3.72, 5.11)	0.044
TG, mmol/L	1.38 (1, 1.99)	1.43 (1.01, 1.98)	0.379
HDL-C, mmol/L	1.15 (0.98, 1.36)	1.07 (0.93, 1.24)	< 0.001
LDL-C, mmol/L	2.64 (2.15, 3.22)	2.88 (2.27, 3.45)	0.001
aAIP	1.07 (0.89, 1.26)	1.13 (0.96, 1.3)	0.007
Non-HDL-C	3.09 (2.48, 3.69)	3.32 (2.64, 3.99)	< 0.001
LDL-C/HDL-C	2.28 (1.75, 2.96)	2.66 (2.09, 3.34)	< 0.001
AI	2.6 (2.04, 3.4)	3.05 (2.38, 3.91)	< 0.001
LCI	13.42 (7.69, 25.23)	16.77 (9.3, 30.58)	< 0.001

CAD coronary atherosclerotic disease, BMI body mass index, PH primary hypertension, T2DM type 2 diabetes mellitus, SBP systolic blood pressure, DBP diastolic blood pressure, HR heart rate, BPM beats per minute, WBC white blood cell, PLT platelet, eGFR estimated glomerular filtration rate, SUA serum uricacid, TC total cholesterol, TG triglyceride, HDL-C high-density lipoprotein cholesterol, LDL-C low-density lipoprotein cholesterol, aAIP adjusted atherogenic index of plasma, non-HDL-C non-high-density lipoprotein cholesterol, AI atherogenic index, LCI lipoprotein combine index

### Factors influencing aAIP

**Fig. 3:**
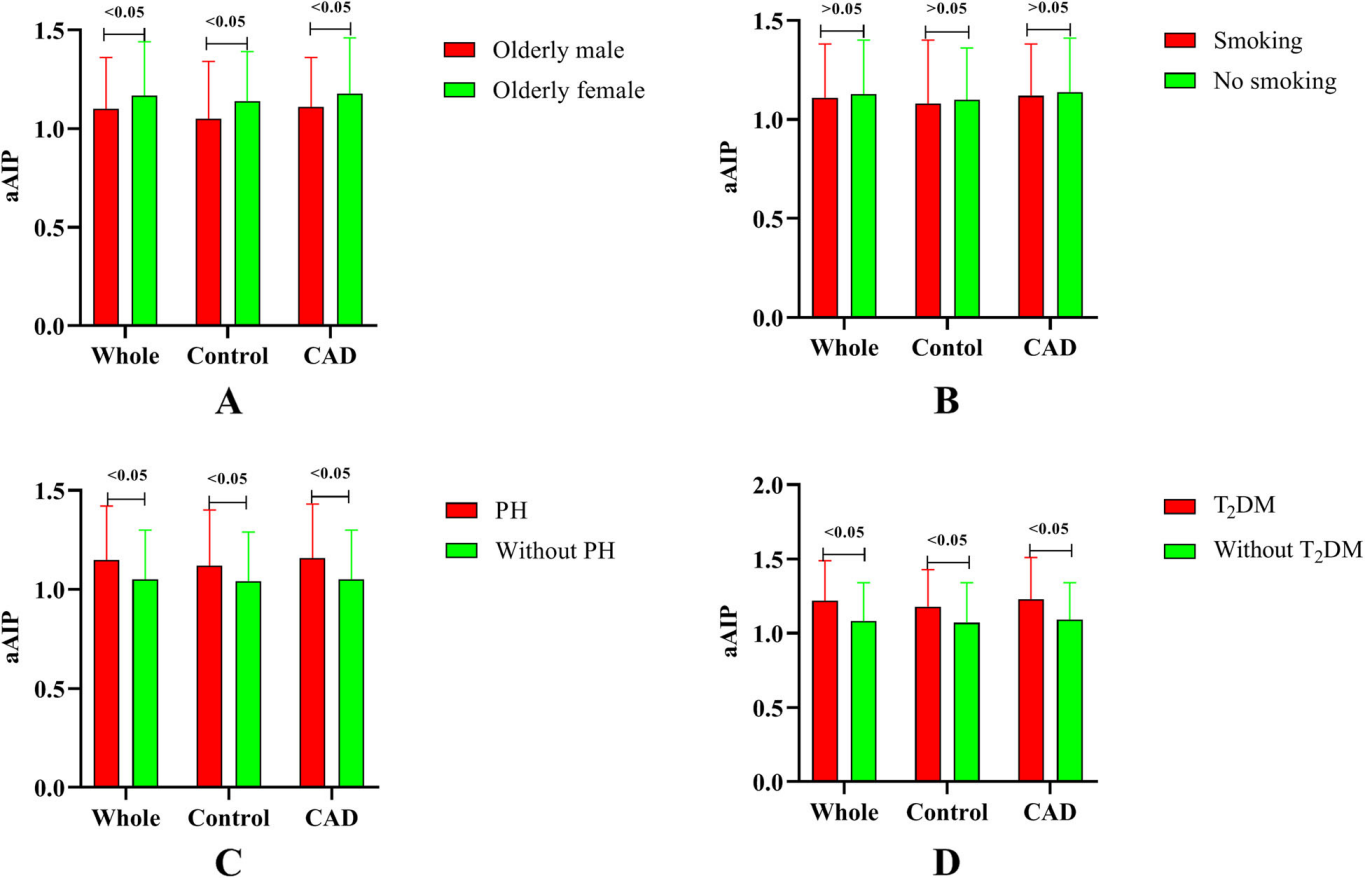
Influence factors of aAIP. A, Gender; B, Smoking; C, PH; D, T2DM

**Fig. 4:**
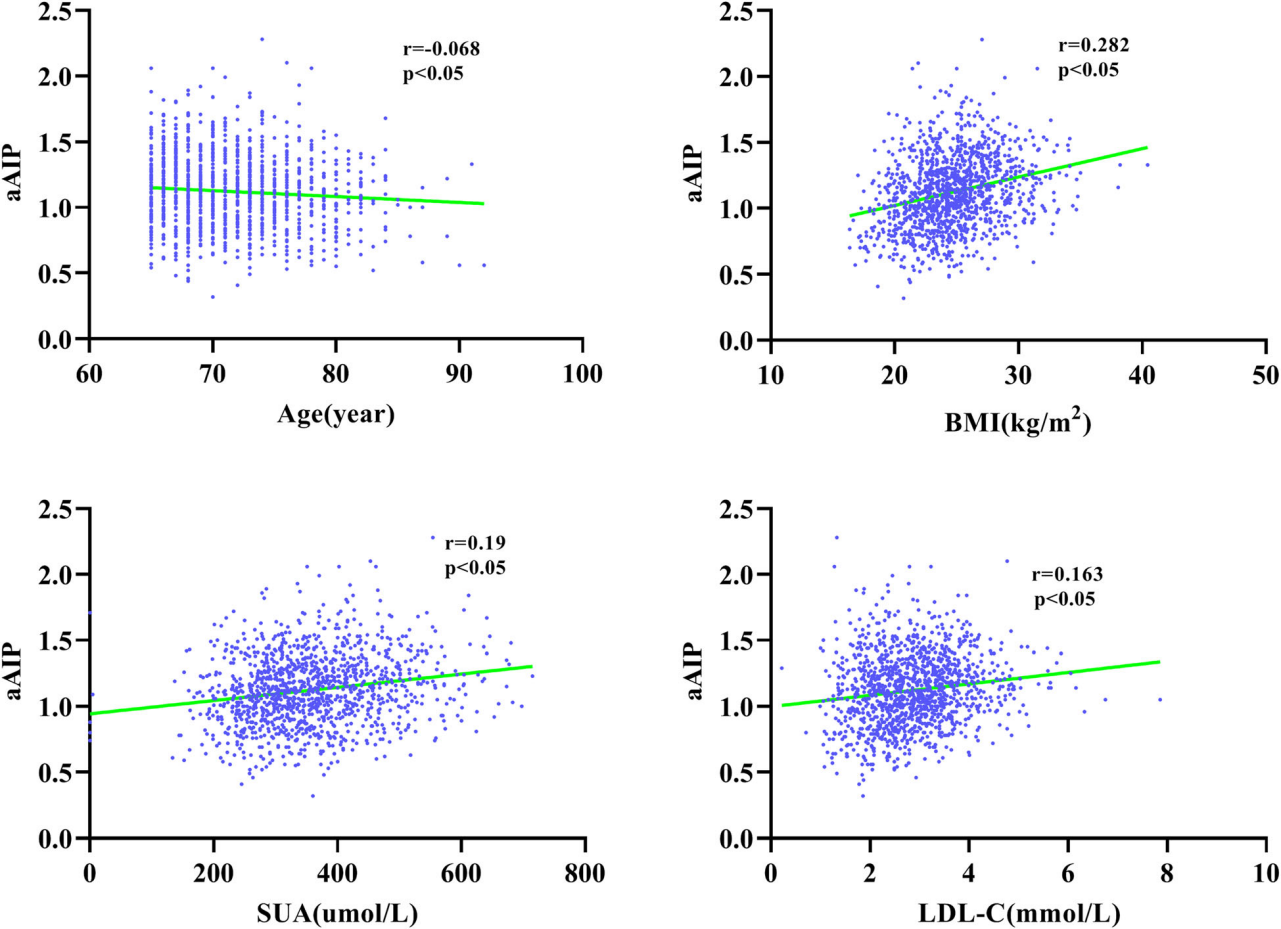
Linear correlation analysis of aAIP with age, BMI, SUA, and LDL-C

As shown in Fig. 3, in both the CAD and control groups, aAIP was higher in females, and subjects with PH or T2DM also had higher levels of aAIP than those without (all *P* < 0.05). However, it was surprising that the aAIP levels were not significantly different in smoking and non-smoking individuals. As shown in Fig. 4, Spearman correlation analysis indicated that aAIP was significantly positively associated with BMI, SUA and LDL-C. However, aAIP was negatively associated with age (all *P* < 0.05).

As shown in Table 2, all variables were compared in four aAIP levels based on median and IQR. The results revealed that the proportion of CAD, males, drinkers, PH, and T2DM and the levels of BMI, SBP, DBP, PLT, eGFR, SUA, TC, LDL-C, HDL-C, TG, non-HDL-C, LDL-C/HDL-C, AI and LCI were significantly different at the four levels of aAIP (all *P* < 0.05).

**Table 2:** Clinical Characteristics in four different aAIP levels

Characteristics	aAIP < 0.94(***n*** = 321)	0.94 ≤ aAIP < 1.12(***n*** = 335)	1.12 ≤ aAIP < 1.285(***n*** = 329)	aAIP ≥ 1.285(***n*** = 328)	***P***
CAD, n(%)	214 (66.7)	240 (71.6)	254 (77.2)	251 (76.5)	0.008
Male, n(%)	221 (68.8)	202 (60.3)	192 (58.4)	178 (54.3)	0.002
Age, year	71 (68, 75)	71 (68, 75)	71 (67, 75)	70 (67, 73.75)	0.085
BMI, kg/m^2^	23.36 (21.09, 25.32)	24.22 (22.49, 26.04)	24.74 (22.86, 26.67)	25.47 (23.44, 27.66)	< 0.001
Smoker, n(%)	105 (32.7)	105 (31.3)	93 (28.3)	99 (30.2)	0.653
Drinker, n(%)	50 (15..6)	31 (9.3)	44 (13.4)	28 (8.5)	0.013
PH, n(%)	221,567)	239 (71.3)	263 (79.9)	279 (85.1)	< 0.001
T2DM, n(%)	49 (15.3)	89 (26.6)	116 (35.3)	138 (42.1)	< 0.001
SBP, mmHg	136 (124.5, 150)	140 (123, 150)	140 (130, 152.5)	140 (130, 152)	0.01
DBP, mmHg	80 (70, 86.5)	80 (72, 86)	80 (73, 89.5)	81 (74, 89.75)	0.012
HR, BPM	72 (65, 80)	72 (66, 80)	72 (68, 81)	72 (68, 80)	0.26
Laboratory parameters					
WBC, 10^9^ /L	6.24 (5.05, 7.94)	6.46 (5.36, 8.07)	6.49 (5.41, 8.03)	6.68 (5.65, 8.02)	0.071
PLT, 10^9^ /L	183.5 (150, 222.5)	198 (160, 232)	193 (161, 236.5)	202 (169, 238.75)	< 0.001
eGFR, ml/min	71.19 (59.95, 85.55)	73.48 (60.87, 86.81)	74.62 (61.01, 87.66)	77.62 (64.87, 90.83)	0.008
SUA, umol/L	325.05 (268.25, 390)	334.3 (273, 400)	365 (300, 430)	368 (309.6, 437.15)	< 0.001
TC, mmol/L	4.16 (3.56, 4.78)	4.36 (3.71, 5.01)	4.45 (3.7, 5.23)	4.62 (3.89, 5.31)	< 0.001
TG, mmol/L	0.85 (0.7, 0.97)	1.22 (1.05, 1.38)	1.65 (1.45, 1.89)	2.57 (2.14, 3.31)	< 0.001
HDL-C, mmol/L	1.33 (1.16, 1.52)	1.14 (1.01, 1.28)	1.04 (0.93, 1.15)	0.94 (0.82, 1.04)	< 0.001
LDL-C, mmol/L	2.51 (2.04, 3.07)	2.93 (2.33, 3.39)	3.03 (2.32, 3.61)	2.9 (2.3, 3.52)	< 0.001
Non-HDL-C	2.76 (2.30, 3.37)	3.23 (2.62, 3.76)	3.39 (2.70, 4.10)	3.66 (2.97, 4.31)	< 0.001
LDL-C/HDL-C	1.89 (1.48, 2.33)	2.48 (2.03, 3.04)	2.85 (2.25, 3.48)	3.12 (2.53, 3.66)	< 0.001
AI	2.12 (1.68, 2.56)	2.74 (2.32, 3.36)	3.27 (2.59, 3.95)	3.88 (3.28, 4.52)	< 0.001
LCI	6.53 (4.13, 9.25)	13.4 (9.25, 18.23)	20.49 (13.75, 30.32)	38.4 (24.64, 56.9)	< 0.001

CAD coronary atherosclerotic disease, BMI body mass index, PH primary hypertension, T2DM type 2 diabetes mellitus, SBP systolic blood pressure, DBP diastolic blood pressure, HR heart rate, BPM beats per minute, WBC white blood cell, PLT platelet, eGFR estimated glomerular filtration rate, SUA serum uricacid, aAIP adjusted atherogenic index of plasma, TC total cholesterol, TG triglyceride, HDL-C high-density lipoprotein cholesterol, LDL-C low-density lipoprotein cholesterol, non-HDL-C non-high-density lipoprotein cholesterol, AI atherogenic index, LCI lipoprotein combine index

### Subgroup analyses

Subgroup analyses showed that clinical characteristics differed between the two sexes. Among elderly males, individuals with CAD had higher PLT and aAIP levels but had a lower prevalence of alcohol consumption and a lower level of SUA than those of the controls. In elderly females, no differences were noted between the two groups. In contrast, the prevalence of PH and the levels of age, SBP, HR and TC were higher in CAD individuals than in the controls (Table 3 in supply materials).

### Multivariate logistic regression analysis

**Fig. 5:**
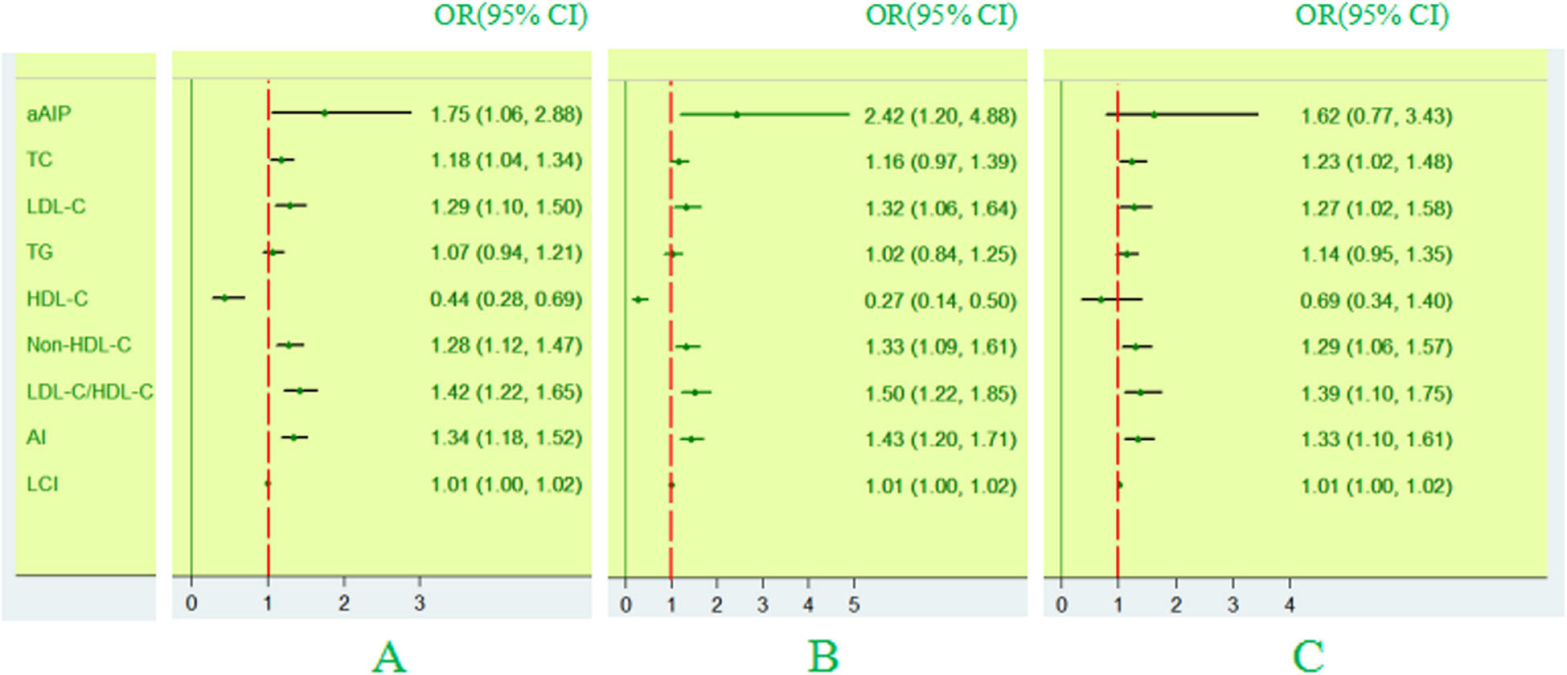
Forest plot of multivariate logistic regression analyses of CAD risk factors. A, in whole population; B, in elderly males; C, in elderly females

Multivariate logistic regression analyses were executed in all subjects, elderly males and elderly females to identify the risk factors for CAD. As represented in Fig. 5, aAIP remained a risk factor for CAD in elderly individuals after adjusting for sex, age, smoking status, pH, T2DM, HR, WBC and PLT (OR 1.75; 95% CI: 1.06–2.88, *P* < 0.05) and was superior to traditional or other nontraditional lipid indices. In subgroup analyses, aAIP remained a dependent risk factor for CAD in elderly males after adjusting for confounding factors (OR 2.42; 95% CI: 1.2–4.88, *P* < 0.05). Conversely, in the elderly female model, aAIP was not an independent risk factor for CAD after adjusting for confounding factors (*P* > 0.05) (Fig. 5). Further more, a sensitivity analysis excluding coronary emergency showed a similar results (Table 4 in supply materials).

## Discussion

The present study discovered that AIP was independently and positively related to the presence and severity of CAD in elderly individuals and was superior to traditional and other nontraditional lipid indices. However, subgroup analyses did not supply any evidence to support this conclusion in elderly females.

Coronary atherosclerosis is the pathological basis of CAD, and lipid deposition is the leading cause of coronary atherosclerosis. Among them, LDL-C is a chief causal factor [17], and its cumulative exposure is a larger risk factor for CAD, especially in elderly individuals, even independent of a single LDL-C level [18]. Accordingly, decreasing LDL-C levels can reduce cardiovascular events [19, 20].

As reported in a previous study, small dense low-density lipoprotein (sdLDL) is a component of LDL particles with a small volume and high density [21]. Compared to other LDL, sdLDL is more easily oxidized and damages vascular endothelial cells. Then, sdLDL passes through cells, resulting in cholesterol deposition and ultimately lead to atherosclerosis [22]. As a result, lipid-induced atherosclerosis could be comprehensively assessed by combining routine blood lipids and sdLDL [23]. Nevertheless, the procedure of testing sdLDLs is so complex and expensive that it cannot be widely used in clinical practice. Therefore, we need to identify a simple method to indirectly measure LDL particle size.

Dobiásová et al. [24] introduced the conception of AIP first in 2001, reporting that it had better clinical application value than the single traditional lipid index. Subsequently, an increasing amount of evidence showed that AIP was negatively correlated with LDL particle size and can generally indicate sdLDL size [25]. Afterwards, AIP was gradually defined to be related to T2DM [26], abdominal obesity [27], non-alcoholic fatty liver [28], arterial stiffness [29] and other factors. Recently, a hospital-based study involving 3600 individuals who underwent CAG reported that AIP could reliably diagnose CAD with a sensitivity of 76.4% and specificity of 61.8% [30]. Furthermore, another study found that AIP could be regarded as a cardiovascular risk factor in Mexicans aged 18 to 22 years [31], which was similar to findings from a previous study in China [9].

However, the correlation between AIP and CAD in elderly individuals (age ≥ 65 years) has not been well established. Moreover, whether AIP can reliably predict the risk of CAD in elderly females has been controversial. On the one hand, a study including 4644 postmenopausal women suggested that AIP might independently predict CAD in postmenopausal women [11]. On the other hand, Nansseu JR et al. [12] found that AIP might not independently impact the risk of CAD among Cameroonian postmenopausal women. Similarly, another study reported that AIP did not significantly increase the risk of acute coronary syndrome in females (average age 64.9 ± 10 years) [32]. Therefore, this study analysed the association between AIP and the risk of CAD in elderly individuals, a special group who were at high risk of having CAD. In addition, because the role of AIP in predicting CAD in elderly females is controversial, subgroup analysis by sex was conducted to evaluate the correlation between AIP and CAD.

Why does the difference in the relationship between AIP and CAD still exist in the two sexes, even though females in this age stage are minimally influenced by oestrogen? Several factors must be taken into account to answer this question. First, Fig. 3 shows that median AIP levels in elderly females were significantly higher than that in males, which might play an important role in accounting for this difference. In addition, PLT levels, which might influence the level of AIP according to Table 2, differed between the CAD group and controls in elderly males rather than elderly females. Indeed, some physical data, such as exercise and diet, that influence TG or HDL-C could not be acquired in this study, which may confound the results.

In addition, this study found that the levels of SUA, BMI, WBC, PLT, eGFR, LDL-C and other lipid indices and the proportion of patients with PH and T2DM was closely related to AIP, and all of these factors were considered risk factors for CAD in a previous study [6, 33–36]. In view of this relationship, both elderly males and females should be encouraged to adhere to a healthy lifestyle to reduce AIP levels, even if the association between AIP and CAD risk was not established in elderly females.

### Strength

This present study has some following strengths. First, the subjects of this study were elderly individuals (age ≥ 65 years), a group for whom controversy exists regarding the relationship between AIP and CAD risk. This study calculated aAIP levels in elderly individuals with different numbers of main coronary artery stenoses. Then, the correlation between aAIP and other risk factors for CAD was also analysed. Moreover, subgroup analysis stratified by sex was conducted.

### Limitation

Some limitations have to be taken into account. First, due to the cross-sectional and retrospective design, the cause-result effect cannot be well interpreted. In the future, prospective and cohort studies should be conducted to verify the results. Second, as a retrospective study, some data related to AIP, such as waist circumference, diet and physical labour, could not be acquired in the analysis, which might interfere with the final results. Furthermore, the individuals included in controls were not truly healthy, and their representativeness needs to be further verified. Finally, the Gensini score could not be used to assess the severity of coronary stenosis. However, the number of diseased coronary arteries was used to evaluate the correlation between AIP and the severity of CAD according to the medical records.

## Conclusion

AIP was independently and positively related to the risk and severity of CAD in elderly males but not in elderly females, which might be superior to traditional and other nontraditional lipid profiles. In the future, AIP may be used in the clinic to predict and prevent CAD as early as possible in elderly males, especially those with risk factors for CAD.

## Supplementary Information

**Additional file 1:.** Table S3 Clinical characteristics in elderly males and females. Table S4 Multivariate logistic regression analysis of individuals after excluding coronary emergency.

## Data Availability

The datasets of this study are available from the corresponding author.

## Abbreviations

CAD: coronary atherosclerotic disease
CAG: coronary angiography
BMI: body mass index
PH: primary hypertension
T2DM: Type 2 diabetes mellitus
HR: heart rate
BPM: beats per minute
SBP: systolic blood pressure
DBP: diastolic blood pressure
WBC: white blood cell
ALT: alanine aminotransferase
eGFR: estimated glomerular filtration rate
PLT: platelet
SUA: serum uricacid
aAIP: adjusted atherogenic index of plasma
TC: total cholesterol
HDL-C: high-density lipoprotein cholesterol
non-HDL-C: non-high-density lipoprotein cholesterol
LDL-C: low-density lipoprotein cholesterol
TG: triglyceride
AI: atherogenic index
LCI: lipoprotein combine index
OR: odds ratio
95% CI: 95% confidence interval
IQR: interquartile range
