# Supplementary material for: Atherogenic index of plasma is related to coronary atherosclerotic disease in elderly individuals: a cross-sectional study
Source: Lipids Health Dis. 2021 Jul 11;20:68. doi: 10.1186/s12944-021-01496-8 (PMC8273949; doi:10.1186/s12944-021-01496-8)
Supplement: Supplementary file 1 — Additional file 1:. Table S3 Clinical characteristics in elderly males and females. Table S4 Multivariate logistic regression analysis of individuals after excluding coronary emergency. [file 12944_2021_1496_MOESM1_ESM.docx]

**Table 3** Clinical characteristics in elderly males and females

| **Characteristics** | **Male(n=793)** | | | **Female(n=520)** | | |
| --- | --- | --- | --- | --- | --- | --- |
|  | **Control(n=183)** | **CAD(n=610)** | ***P*** | **Control(n=171)** | **CAD(n=349)** | ***P*** |
| Age, year | 70(67, 74) | 71(68, 74) | 0.176 | 70(67, 73) | 71(68, 75.5) | 0.001 |
| BMI, kg/m^2^ | 24.34(22.49, 26.40) | 24.22(22.27, 26.15) | 0.434 | 24.89(22.96, 28.13) | 24.77(22.6, 26.98) | 0.166 |
| Smoker, n(%) | 85(46.4) | 312(51.1) | 0.265 | 1(0.6) | 4(1.1) | 0.538 |
| Drinker, n(%) | 44(24) | 107(17.5) | 0.049 | 1(0.6) | 1(0.3) | 0.606 |
| PH, n(%) | 129(70.5) | 459(75.2) | 0.198 | 119(69.6) | 289(82.8) | 0.001 |
| T2DM, n(%) | 31(16.9) | 180(29.5) | 0.001 | 48(28.1) | 133(38.1) | 0.024 |
| SBP, mmHg | 139(127, 148) | 138(125, 150) | 0.896 | 139(129, 150) | 141(130, 156) | 0.022 |
| DBP, mmHg | 80(70, 87) | 80(72, 89) | 0.416 | 80(73, 86) | 80(73, 89) | 0.253 |
| HR, BPM | 70(65, 79) | 72(66, 80) | 0.142 | 70(67, 80) | 74(68, 82) | 0.049 |
| Laboratory parameters |  |  |  |  |  |  |
| WBC, 10^9^ /L | 6.24(4.88, 7.33) | 6.96(5.58, 8.78) | <0.001 | 5.79(5.02, 6.64) | 6.42(5.42, 8.04) | <0.001 |
| PLT, 10^9^ /L | 177(146, 213) | 190(157, 228.5) | 0.001 | 201(166, 241) | 207(169.5, 244) | 0.389 |
| eGFR, ml/min | 76.75(64.52, 91.09) | 76.35(61.98, 89.92) | 0.408 | 73.57(61.49, 87.15) | 70.34(59.33, 82.45) | 0.084 |
| SUA, umol/L | 385(325.53, 461.08) | 355.6(297.9, 430.4) | 0.001 | 310(257.5, 389.7) | 323(270, 384.75) | 0.237 |
| TC, mmol/L | 4.1(3.55, 4.66) | 4.24(3.54, 4.91) | 0.075 | 4.57±1.04 | 4.83±1.09 | 0.011 |
| TG, mmol/L | 1.21(0.87, 1.73) | 1.32(0.96, 1.78) | 0.094 | 1.54(1.15, 2.15) | 1.63(1.21, 2.28) | 0.317 |
| HDL-C, mmol/L | 1.12(0.95, 1.35) | 1.04(0.9, 1.19) | <0.001 | 1.20(1.02, 1.39) | 1.13(0.98, 1.3) | 0.021 |
| LDL-C, mmol/L | 2.52(2.06, 3.03) | 2.78(2.18, 3.33) | 0.001 | 2.87±0.86 | 3.11±0.92 | 0.005 |
| aAIP | 1.05±0.28 | 1.11±0.26 | 0.011 | 1.11(0.96, 1.28) | 1.17(1, 1.34) | 0.096 |
| Non-HDL-C | 2.85(2.36, 3.43) | 3.14(2.49, 3.79) | 0.001 | 3.33(2.96, 4.05) | 3.61(2.91, 4.25) | 0.002 |
| LDL-C/HDL-C | 2.24(1.72, 2.89) | 2.66(2.07, 3.30) | <0.001 | 2.31(1.81, 3.04) | 2.65(2.12, 3.36) | <0.001 |
| AI | 2.55(1.88, 3.29) | 3.04(2.33, 3.88) | <0.001 | 2.68(2.12, 3.58) | 3.1(2.45, 3.93) | <0.001 |
| LCI | 10.93(6.43, 19.41) | 14.69(8, 27.26) | <0.001 | 16.79(8.95, 33.45) | 21.01(12.04, 36.86) | 0.006 |

CAD coronary atherosclerotic disease, BMI body mass index, PH primary hypertension, T2DM type 2 diabetes mellitus, SBP systolic blood pressure, DBP diastolic blood pressure, HR heart rate, BPM beats per minute, WBC white blood cell,PLT platelet, eGFR estimated glomerular filtration rate, SUA serum uricacid, TC total cholesterol, TG triglyceride, HDL-C high-density lipoprotein cholesterol, LDL-C low-density lipoprotein cholesterol, aAIP adjusted atherogenic index of plasma, non-HDL-C non-high-density lipoprotein cholesterol, AI atherogenic index, LCI lipoprotein combine index.

**Table 4** Multivariate logistic regression analysis of individuals after excluding coronary emergency.

| Variate | Whole(n=966) | | Elderly male (n=543) | | Elderly female(n=423) | |
| --- | --- | --- | --- | --- | --- | --- |
|  | OR(95% CI) | *P* | OR(95% CI) | *P* | OR(95% CI) | *P* |
| aAIP | 1.99(1.17, 3.40) | 0.012 | 2.53(1.20, 5.32) | 0.014 | 2.05(0.92, 4.59) | 0.081 |
| TC | 1.15(1.00, 1.32) | 0.049 | 1.15(0.94, 1.40) | 0.177 | 1.17(0.97, 1.42) | 0.108 |
| TG | 1.11(0.97, 1.26) | 0.126 | 1.05(0.86, 1.29) | 0.614 | 1.18(0.98, 1.42) | 0.08 |
| HDL-C | 0.48(0.30, 0.79) | 0.003 | 0.29(0.15, 0.57) | <0.001 | 0.66(0.32, 1,37) | 0.263 |
| LDL-C | 1.20(1.02, 1.42) | 0.029 | 1.29(1.02, 1.64) | 0.034 | 1.16(0.92, 1.46) | 0.221 |
| Non-HDL-C | 1.24(1.07, 1.44) | 0.004 | 1.32(1.06, 1.63) | 0.011 | 1.23(1.00, 1.51) | 0.047 |
| LDL-C/HDL-C | 1.34(1.14, 1.58) | <0.001 | 1.48(1.18, 1.86) | 0.001 | 1.3(1.02, 1.65) | 0.032 |
| AI | 1.31(1.14, 1.49) | <0.001 | 1.43(1.18, 1.73) | <0.001 | 1.30(1.07, 1.57) | 0.009 |
| LCI | 1.01(1.00, 1.02) | 0.021 | 1.01(1.00, 1.02) | 0.083 | 1.01(1.00, 1.02) | 0.07 |

In model of whole populations, OR was adjusted for gender, age, PH, T_2_DM, WBC and PLT; In model of elderly males, OR was adjusted for T_2_DM, PLT, SUA; In model of elderly females, OR was adjusted for age, PH, T_2_DM and WBC.

CAD coronary artery disease, aAIP adjusted atherogenic index of plasma, TC total cholesterol, TG triglyceride, LDL-C low-density lipoprotein cholesterol, HDL-C high density lipoprotein cholesterol, non-HDL-C non-high-density lipoprotein cholesterol, AI atherogenic index, LCI lipoprotein combine index.
